# Supplementary material for: Employment Status and Alcohol-Attributable Mortality Risk—A Systematic Review and Meta-Analysis
Source: Int J Environ Res Public Health. 2022 Jun 15;19(12):7354. doi: 10.3390/ijerph19127354 (PMC9224380; doi:10.3390/ijerph19127354)
Supplement: Supplementary file 1 [file ijerph-19-07354-s001.zip › ijerph-1694942-supplementary.pdf]

International Journal of
Environmental Research and Public Health

MDPI

Review

Employment Status and Alcohol-Attributable Mortality Risk—A Systematic Review and Meta-Analysis

Celine Saul[1], Shannon Lange[2-4] and Charlotte Probst[1-4,*]

[1] Heidelberg Institute of Global Health (HIGH), Medical Faculty and University Hospital, Heidelberg University, Heidelberg, Germany
[2] Institute for Mental Health Policy Research, Centre for Addiction and Mental Health (CAMH), Toronto, ON, Canada
[3] Department of Psychiatry, University of Toronto, Toronto, ON, Canada
[4] Campbell Family Mental Health Research Institute, CAMH, Toronto, Ontario, Canada
* Correspondence: Charlotte Probst, Institute for Mental Health Policy Research, Centre for Addiction and Mental Health (CAMH), 33 Ursula Franklin Street, Toronto ON, Canada M5S 2S1, Tel.: +1 416 535 8501 ext. 36510, E-Mail: mariecharlotte.probst@gmail.com

This file includes:

Table S1: PRISMA 2009 checklist.

Text S1: Search terms.

Table S2: Diagnoses and ICD-10 codes of 100% alcohol-attributable condition.

Table S3: Diagnoses and ICD-10 codes of conditions with an alcohol-attributable fraction >10% for mortality globally.

Table S4: Inclusion criteria.

Table S5: Quality rating.

Table S6: Quality checklist. Ratings on population representativeness of the sample, measurement of socioeconomic status (SES), operationalization of alcohol-attributable mortality, data linkage, and age-adjustment for each study included in the meta-analysis.

Table S7: Causes of death included in each of the studies.

Table S1. PRISMA 2009 checklist [1].

Section/topic	#	Checklist item	Reported on page #
**TITLE**			
Title	1	Identify the report as a systematic review, meta-analysis, or both.	Title page
**ABSTRACT**			
Structured summary	2	Provide a structured summary including, as applicable: background; objectives; data sources; study eligibility criteria, participants, and interventions; study appraisal and synthesis methods; results; limitations; conclusions and implications of key findings; systematic review registration number.	Title page
**INTRODUCTION**			
Rationale	3	Describe the rationale for the review in the context of what is already known.	1-2
Objectives	4	Provide an explicit statement of questions being addressed with reference to participants, interventions, comparisons, outcomes, and study design (PICOS).	2
**METHODS**			
Protocol and registration	5	Indicate if a review protocol exists, if and where it can be accessed (e.g., Web address), and, if available, provide registration information including registration number.	2
Eligibility criteria	6	Specify study characteristics (e.g., PICOS, length of follow-up) and report characteristics (e.g., years considered, language, publication status) used as criteria for eligibility, giving rationale.	2-3
Information sources	7	Describe all information sources (e.g., databases with dates of coverage, contact with study authors to identify additional studies) in the search and date last searched.	2
Search	8	Present full electronic search strategy for at least one database, including any limits used, such that it could be repeated.	Text S1
Study selection	9	State the process for selecting studies (i.e., screening, eligibility, included in systematic review, and, if applicable, included in the meta-analysis).	2-3, Figure 1
Data collection process	10	Describe method of data extraction from reports (e.g., piloted forms, independently, in duplicate) and any processes for obtaining and confirming data from investigators.	3
Data items	11	List and define all variables for which data were sought (e.g., PICOS, funding sources) and any assumptions and simplifications made.	3

Section/topic	#	Checklist item	Reported on page #
Risk of bias in individual studies	12	Describe methods used for assessing risk of bias of individual studies (including specification of whether this was done at the study or outcome level), and how this information is to be used in any data synthesis.	3
Summary measures	13	State the principal summary measures (e.g., risk ratio, difference in means).	3
Risk of bias across studies	15	Specify any assessment of risk of bias that may affect the cumulative evidence (e.g., publication bias, selective reporting within studies).	3
Synthesis of results	14	Describe the methods of handling data and combining results of studies, if done, including measures of consistency (e.g., I^2) for each meta-analysis.	3
Additional analyses	16	Describe methods of additional analyses (e.g., sensitivity or subgroup analyses, meta-regression), if done, indicating which were pre-specified.	n.a.
**RESULTS**			
Study selection	17	Give numbers of studies screened, assessed for eligibility, and included in the review, with reasons for exclusions at each stage, ideally with a flow diagram.	3, Figure 1
Study characteristics	18	For each study, present characteristics for which data were extracted (e.g., study size, PICOS, follow-up period) and provide the citations.	4, Table 1
Risk of bias within studies	19	Present data on risk of bias of each study and, if available, any outcome level assessment (see item 12).	6, Figure 2
Results of individual studies	20	For all outcomes considered (benefits or harms), present, for each study: (a) simple summary data for each intervention group (b) effect estimates and confidence intervals, ideally with a forest plot.	Figure 2, Table 1 as applicable
Synthesis of results	21	Present results of each meta-analysis done, including confidence intervals and measures of consistency.	6, Figure 2
Risk of bias across studies	22	Present results of any assessment of risk of bias across studies (see Item 15).	6
Additional analysis	23	Give results of additional analyses, if done (e.g., sensitivity or subgroup analyses, meta-regression [see Item 16]).	6

Section/topic	#	Checklist item	Reported on page #
**DISCUSSION**			
Summary of evidence	24	Summarize the main findings including the strength of evidence for each main outcome; consider their relevance to key groups (e.g., healthcare providers, users, and policy makers).	7
Limitations	25	Discuss limitations at study and outcome level (e.g., risk of bias), and at review-level (e.g., incomplete retrieval of identified research, reporting bias).	8
Conclusions	26	Provide a general interpretation of the results in the context of other evidence, and implications for future research.	8
**FUNDING**			
Funding	27	Describe sources of funding for the systematic review and other support (e.g., supply of data); role of funders for the systematic review.	8

From: Moher D, Liberati A, Tetzlaff J, Altman DG, The PRISMA Group (2009). Preferred Reporting Items for Systematic Reviews and Meta-Analyses: The PRISMA Statement. PLoS Med 6(7): e1000097. doi:10.1371/journal.pmed1000097

For more information, visit: www.prisma-statement.org.

Text S1. Search terms.

The searches included a set of keywords, wildcards, truncation and medical subject headings (where applicable). The search terms used are shown below.

OvidSP (Embase, MEDLINE, and PsycINFO)

(alcohol *abuse/ OR (alcohol abuse).ti,ab. OR exp *Drinking Behavior/ OR exp *Alcohol Drinking/ OR exp *Drinking/ OR *Binge Drinking/ OR (alcoholic beverages).ti,ab. OR (alcohol and (drinking or intake or consumption)).ti,ab.)
AND
(exp *socioeconomic factors/ OR exp *social class/ OR socio?economic status.ti,ab. OR Educational status/ OR income/ OR Employment/ OR (education* ADJ (level OR attain*)).ti,ab. OR (socio?economic OR SES OR SEP OR asset? score OR income).ti,ab.)
AND
(exp Mortality/ OR exp Mortality, Premature/ OR exp *excess mortality/ OR *differential mortality/ OR (mortality).ti,ab. OR *cause of death/)
AND
(exp Case?Control Studies/ OR exp Cohort studies/ OR (case OR cohort OR control group* OR ratio OR risk* OR prospective* OR follow* OR longitudinal OR retrospective OR effect modifi*).ti,ab.)
Limit 1 to yr="2013-Current"

Web of Science

TS=(alcoholic beverages OR alcohol AND (drinking OR intake OR consumption)) OR TS=(Alcohol abuse)
AND
TS=(mortality OR death* OR cause of death)
AND
TS=(ses OR socioe$conomic status OR social class OR socio$economic variable* OR (education* AND (attain* OR status OR level)) OR income OR employment)
AND
TS=(case?control stud* OR cohort?stud* OR ratio OR risk* OR prospective* OR follow* OR longitudinal OR retrospective OR effect modifi*)

Table S2. Diagnoses and ICD-10 codes of 100% alcohol-attributable conditions [2].

Diagnosis	ICD-10 code
Alcohol-induced pseudo-Cushing's syndrome	E24.4
Mental and behavioural disorders due to use of alcohol	F10
Acute intoxication	F10.0
Harmful use	F10.1
Dependence syndrome	F10.2
Withdrawal state	F10.3
Withdrawal state with delirium	F10.4
Psychotic disorder	F10.5
Amnesic syndrome	F10.6
Residual and late-onset psychotic disorder	F10.7
Other mental and behavioural disorders	F10.8
Unspecified mental and behavioural disorder	F10.9
Degeneration of nervous system due to alcohol	G31.2
Alcoholic polyneuropathy	G62.1
Alcoholic myopathy	G72.1
Alcoholic cardiomyopathy	I42.6
Alcoholic gastritis	K29.2
Alcoholic liver disease	K70
Alcoholic fatty liver	K70.0
Alcoholic hepatitis	K70.1
Alcoholic fibrosis and sclerosis of liver	K70.2
Alcoholic cirrhosis of liver	K70.3
Alcoholic hepatic failure	K70.4
Alcoholic liver disease, unspecified	K70.9
Alcohol-induced pancreatitis	K85.2
Alcohol-induced chronic pancreatitis	K86.0
Finding of alcohol in blood	R78.0
Toxic effect of alcohol	T51
Toxic effect of ethanol	T51.0
Toxic effect of methanol	T51.1
Toxic effect of other alcohols	T51.8
Toxic effect of alcohol, unspecified	T51.9
Accidental poisoning by and exposure to alcohol	X45
Intentional self-poisoning by and exposure to alcohol	X65
Poisoning by and exposure to alcohol, undetermined intent	Y15
Evidence of alcohol involvement determined by blood alcohol level	Y90

Table S3. Diagnoses and ICD-10 codes of conditions with an alcohol-attributable fraction >10% for mortality globally [3].

Category	Sub-category	Cause of death	ICD-10 code	AAF
Communicable diseases	Respiratory infections and tuberculosis	Tuberculosis	A15-A19, B90	20%
Injuries	Intentional injuries	Self-harm	X60-64, X66-X69, X65, X70-X84	18%
		Interpersonal violence	X85–Y09, Y871	18%
	Unintentional injuries	Road injury	V01–V04, V06, V09–V80, V87, V89, V99	27%
		Poisonings	X40, X43, X46–X48, X49	12%
		Falls	W00–W19	11%
		Drowning	W65–W74	12%
		Exposure to mechanical forces	W20–W38, W40–W43, W45, W46, W49–W52, W75, W76	14%
		Other unintentional injuries	V2*, W39, W44, W53-W64, W77-W99, X20-X29, X50-X59, Y40-Y86, Y88, Y89	14%
Noncommunicable diseases	Digestive diseases	Cirrhosis of the liver	K70, K74	48%
		Pancreatitis	K85–K86	26%
	Malignant neoplasms	Lip and oral cavity	C00-C08	26%
		Other pharyngeal cancers	C09–C10, C12–C14	31%
		Oesophagus cancer	C15	17%
		Colon and rectum cancers	C18-C21	11%
		Larynx cancer	C32	22%
	Neurological disorders	Epilepsy	G40-G41	13%

* V-series not included in road injuries
AAF alcohol-attributable fraction.
Note. 100% alcohol-attributable conditions are excluded from this table and shown in Table S2.

Table S4. Inclusion criteria.

Criterion	Inclusion	Exclusion
Outcome	The outcome is mortality measured at the individual level	Indirectly affected people are investigated (e.g., sober victims of car drivers under the influence of alcohol)
	The outcome is attributable to alcohol use	
SES	SES is measured via occupation or employment	SES is measured only via income, or education
	SES is measured at the individual, parental, or household level	SES is measured on the neighborhood-level
Design	The study is empirical and quantitative	The study is an intervention study
	The study employed a case-control, cohort/prospective/longitudinal, data-linkage, or retrospective design	The study is using exclusively cross-sectional data
Sample	The sample is population-based	A clinical sample is investigated
	Participants are at least 15 years of age	
Results	The outcome (including N) is reported by SES of the deceased	
	One point estimate measure of relative risk (RR, odds ratio (OR), hazard ratio (HR), standardized mortality ratio (SMR) etc.) comparing risk in different SES strata and its confidence interval, standard error, or sufficient raw data for calculation (N total and n cases) are reported	
Language	No restrictions	
Time	Studies published since February 2013	

Table S5. Quality rating.

Criterion	Categories	Evaluation	Code
Sample representativeness	Nationally representative sample	Satisfactory	0
	Fraction of the target population	Insufficient	1
Assessment of SES	Assigned based on most recent census	Satisfactory	0a
	Assigned through family or household member	Satisfactory	0b
	Use of an own or 'other' category	Satisfactory	0c
	One or more pairwise comparisons are not reported	Satisfactory	0d
	Exclusion of meaningful parts of the population	Insufficient	1
Definition of alcohol-attributable mortality	100% alcohol-attributable mortality exclusively	Satisfactory	0
	Inclusion of diseases of the liver and/or pancreatitis	Insufficient	1a
	Inclusion of neoplasms in the upper gastrointestinal tract	Insufficient	1b
	Inclusion of all types of injures	Insufficient	1c
	Inclusion of two or more of the above categories	Flawed	2
Data linkage	Individual linkage	Satisfactory	0
	No linkage	Insufficient	1
Age adjustment	Age-adjusted	Satisfactory	0
	Not age-adjusted	Insufficient	1

SES, socioeconomic status.

Table S6. Quality checklist. Ratings on population representativeness of the sample, measurement of socioeconomic status (SES), operationalization of alcohol-attributable mortality, data linkage, and age-adjustment for each study included in the meta-analysis.

Study	Population rep-resenta-tiveness of the sample	Measurement of SES	Operationalization of alcohol-attributable mortality	Data linkage	Age adjust-ment
Agren et al., 1992 [4]	0	0	0	0	0
Connolly et al., 2010 [5]	0	0	1	0	0
Grigoriev et al., 2017 [6]	0	0	0	0	0
Herttua et al., 2008 [7]	0	0	0	0	0
Mäki et al., 2008 [8]	0	1	1	0	0
Mäki et al., 2009 [9]	0	1	1	0	0
Mustard et al., 2013 [10]	0	0	0	0	0
Pulido et al., 2017 [11]	0	0	0	0	0
Vågerö & Garcy, 2016 [12]	0	0	0	0	0
Zagozdzon et al., 2009 [13]	0	0	1	1	0

0=Satisfactory, criterion is met (e.g., sample is representative; data were linked etc.)
1=Not satisfactory, criterion is not met

Table S7. Causes of death included in each of the studies.

Study	Includes deaths with an AAF <100%	ICD version(s)	Causes included
Agren et al., 1992 [4]	No	ICD-8	Alcohol-related liver cirrhosis; high alcohol consumption; alcohol intoxication; alcohol psychosis
Connolly et al., 2010 [5]	Yes	ICD-10	Mental and behavioural disorders due to use of alcohol (F10); degeneration of nervous system due to alcohol (G31.2); alcoholic polyneuropathy (G62.1); alcoholic cardiomyopathy (I42.6); alcoholic gastritis (K29.2); alcoholic liver disease (K70); chronic hepatitis, not elsewhere classified (K73); fibrosis and cirrhosis of liver (K74; excluding K74.3-K74.5 biliary cirrhosis); alcohol-induced chronic pancreatitis (K86.0); accidental poisoning by and exposure to alcohol (X45); intentional self-poisoning by and exposure to alcohol (X65); and poisoning by and exposure to alcohol (Y15)
Grigoriev et al., 2017 [6]	No	ICD-10	Cirrhosis of the liver (K70, K74); accidental poisonings by alcohol (X45)
Herttua et al., 2008 [7]	No	ICD-10	Alcohol dependence syndrome (F10.2), other mental and behavioral disorders due to alcohol use (F10.1 and F10.3-10.9), alcoholic cardiomyopathy (I42.6), alcoholic liver disease (K70), and alcoholic diseases of the pancreas (K86.0), as well as a few rarely occurring additional categories (K29.2, G31.2, G40.51, G62.1, and G72.1)
Mäki et al., 2008 [8]	Yes*	ICD-9/ICD-10	Alcohol-related suicides (ICD9: E95.0-E95.9; ICD10: X60-X84 and Y87.0)<br>Defined as alcohol-attributable as follows: "alcoholic intoxication was stated as one of the three contributory causes,"
Mäki et al., 2009 [9]	Yes*	ICD-9/ICD-10	Alcohol-related suicides (ICD9: E95.0-E95.9; ICD10: X60-X84 and Y87.0)<br>Defined as alcohol-attributable as follows: "in order for alcohol to be classified as a contributory cause the pathologist must determine that intoxication indeed played a role in the process that ended in suicide"
Mustard et al., 2013 [10]	No	ICD-9/ICD-10	Mental and behavioural disorders due to use of alcohol (ICD-9: 291, 303, 305.0; ICD-10: F10); alcoholic cardiomyopathy (ICD-9: 425.5; ICD-10: I42.6); alcoholic liver disease (ICD-9: 5710-5713; ICD-10: K70); alcoholic diseases of the pancreas (ICD-9: 5770, 5771; ICD-10: K85, K86.0) Accidental poisoning by and exposure to alcohol (ICD-9: 860; ICD-10: X45)
Pulido et al., 2017 [11]	No	ICD-10	Alcohol-use disorders (F10); alcoholic cardiomyopathy (I42.6); alcoholic liver disease (K70); alcohol-induced pancreatitis (K85.2, K86.0); alcohol poisoning (X45, X65, Y15); other pathologies due to alcohol (E24.4, G31.2, G62.1, G72.1, K29.2, R78.0)
Vågerö & Garcy, 2016 [12]	No	ICD-10	Mental and behavioural disorders due to use of alcohol (F10); degeneration of nervous system due to alcohol (G31.2); alcoholic polyneuropathy (G62.1); alcoholic cardiomyopathy (I42.6); alcoholic gastritis (K29.2); alcoholic liver disease (K70); alcohol-induced chronic pancreatitis (K86.0); toxic effect of alcohol (T51); alcoholic

			myopathy (G72.1), other pathologies due to alcohol (E52, E24.4, P04.3, O35.4, Q86.0, Z50.2, Z71.4, Z72.1, R78.0)
Zagozdzon et al., 2009 [13]	Yes	ICD-10	Cancers (C01-C06, C09, C10, C12-C15, C22, C32); mental and behavioural disorders due to use of alcohol (F10); alcoholic liver disease (K70)

AAF alcohol-attributable fraction.

* Even though all deaths were defined as alcohol-attributable by the pathologies, we did not code this as 100% attributable only based on to the included ICD codes.

References

1. MOHER D., LIBERATI A., TETZLAFF J., ALTMAN D. G., THE PRISMA GROUP. Preferred reporting items for systematic reviews and meta-analyses: The PRISMA statement, International Journal of Surgery 2010: 8: 336-341.
2. REHM J., GMEL G. E., GMEL G., HASAN O. S. M., IMTIAZ S., POPOVA S. et al. The relationship between different dimensions of alcohol use and the burden of disease-an update, Addiction 2017: 112: 968-1001.
3. WORLD HEALTH ORGANIZATION. Global Status Report on Alcohol and Health. In: Geneva S. W. H. O., editor; 2018.
4. AGREN G., ROMELSJÖ A. Mortality in alcohol-related diseases in Sweden during 1971-80 in relation to occupation marital-status and citizenship in 1970, Scand J Soc Med 1992: 20: 134-142.
5. CONNOLLY S., O'REILLY D., ROSATO M., CARDWELL C. Area of residence and alcohol-related mortality risk: a five-year follow-up study, Addiction 2010: 106: 84-92.
6. GRIGORIEV P., JASILIONIS D., STUMBRYS D., STANKŪNIENĖ V., SHKOLNIKOV V. M. Individual- and area-level characteristics associated with alcohol-related mortality among adult Lithuanian males: A multilevel analysis based on census-linked data, PLOS ONE 2017: 12: e0181622.
7. HERTTUA K., MÄKELÄ P., MARTIKAINEN P. Changes in alcohol-related mortality and its socioeconomic differences after a large reduction in alcohol prices: a natural experiment based on register data, Am J Epidemiol 2008: 168: 1110-1118.

8. MÄKI N., MARTIKAINEN P. The Effects of Education, Social Class and Income on Non-alcohol- and Alcohol-Associated Suicide Mortality: A Register-based Study of Finnish Men Aged 25-64, Eur J Population 2008: 24: 385-404.
9. VALTONEN M., LAAKSONEN D. E., LAUKKANEN J., TOLMUNEN T., RAURAMAA R., VIINAMAKI H. et al. Leisure-time physical activity, cardiorespiratory fitness and feelings of hopelessness in men, Bmc Public Health 2009: 9.
10. MUSTARD C. A., BIELECKY A., ETCHES J., WILKINS R., TJEPKEMA M., AMICK B. C. et al. Mortality following unemployment in Canada, 1991–2001, BMC Public Health 2013: 13: 441.
11. PULIDO J., VALLEJO F., ALONSO-LÓPEZ I., REGIDOR E., VILLAR F., DE LA FUENTE L. et al. Directly alcohol-attributable mortality by industry and occupation in a Spanish Census cohort of economically active population, Drug and Alcohol Dependence 2017: 180: 93-102.
12. VÅGERÖ D., GARCY A. M. Does unemployment cause long-term mortality? Selection and causation after the 1992-96 deep Swedish recession, European journal of public health 2016: 26: 778-783.
13. ZAGOZDZON P., ZABORSKI L., EJSMONT J. Survival and cause-specific mortality among unemployed individuals in Poland during economic transition, J Public Health 2009: 31: 138-146.
